# Observational Study of Vaccine Efficacy 24 Years after the Start of Hepatitis B Vaccination in Two Gambian Villages: No Need for a Booster Dose

**DOI:** 10.1371/journal.pone.0058029

**Published:** 2013-03-22

**Authors:** Maimuna Mendy, Ingrid Peterson, Safayet Hossin, Tom Peto, Momodou L. Jobarteh, Adam Jeng-Barry, Mamadi Sidibeh, Abdoulie Jatta, Sophie E. Moore, Andrew J. Hall, Hilton Whittle

**Affiliations:** 1 Medical Research Council Laboratories, The Gambia, Banjul, the Gambia, West Africa; 2 MRC Keneba, MRC Unit The Gambia, Banjul, The Gambia; 3 London School of Hygiene and Tropical Medicine, London, United Kingdom; University of Birmingham, United Kingdom

## Abstract

**Objectives:**

To determine the duration of protection from hepatitis B vaccine given in infancy and early childhood and asses risk factors for HBV infection and chronic infection.

**Methods:**

In 1984 infant HBV vaccination was started in two Gambian villages. Cross sectional serological surveys have been undertaken every 4 years to determine vaccine efficacy. In the current survey 84.6% of 1508 eligible participants aged 1–28 years were tested. A spouse study was conducted in females (aged 14 years and above) and their male partners.

**Results:**

Vaccine efficacy against chronic infection with hepatitis B virus was 95.1% (95% confidence interval 91.5% to 97.1%), which did not vary significantly between age groups or village. Efficacy against infection was 85.4% (82.7% to 87.7%), falling significantly with age. Concentrations of hepatitis B antibody fell exponentially with age varying according to peak response: 20 years after vaccination only 17.8% (95% CI 10.1–25.6) of persons with a low peak response (10–99 mIU/ml) had detectable HBs antibody compared to 27% (21.9% to 32.2%) of those with a high peak response (>999 mIU/ml). Time since vaccination and a low peak response were the strongest risk factors for HBV infections; males were more susceptible, marriage was not a significant risk for females. Hepatitis B DNA was not detected after infection, which tested soley core antibody positive. An undetectable peak antibody response of <10 mIU/ml and a mother who was hepatitis B e antigen positive were powerful risk factors for chronic infection.

**Conclusions:**

Adolescents and young adults vaccinated in infancy are at increased risk of hepatitis B infection, but not chronic infection. Married women were not at increased risk. There is no compelling evidence for the use of a booster dose of HBV vaccine in The Gambia.

## Introduction

Hepatitis B virus (HBV) is the leading cause of viral hepatitis in humans. About 2 billion people worldwide have been infected with HBV and over 50 million new cases are diagnosed annually. Over 350 million have become chronic carriers of the virus, 60 million of them residing in Africa. According to World Health Organisation, 600,000 persons die each year due to the acute or chronic consequences of hepatitis B [1]–[4]. Transmission in highly endemic areas is primarily horizontal between young children [5]. and less frequently from mother to child [6] whereas in low endemic areas transmission is either through sexual contact or through the use of contaminated needles [7], [8].

HBV is a major cause of liver disease and is strongly associated with the development of hepatocellular carcinoma (HCC) [9]. The majority of children infected perinatally become chronic carriers [10] as do 15–20% of persons infected in early childhood [5], [11]. Approximately one third of HBV carriers will progress to cirrhosis and 25% will develop HCC which is the leading cause of cancer in males in The Gambian and causes between 10–15% of adult male deaths [12].

HBV immunization has been available since 1982 and in 1992, the WHO recommended that childhood HBV vaccination be included in national immunization programs [13]. This is the first vaccine against a major human cancer and has been proved to be effective in preventing HBV infection and its chronic consequences [11], [13]–[15].

After baseline surveys of HBV infection in 1980 and 1984 a programme of HBV immunisation commenced in the villages of Keneba and Manduar villages in the rural West Kiang region of The Gambia [5], [11]. Serological surveys have been conducted every 4–5 years over 24 years to determine vaccine efficacy against infection and chronic infection. This community cohort of persons given HBV in infancy is the largest to date in sub Saharan Africa and has the longest follow-up in the world. The main aim has been to determine the long term efficacy of infant HBV vaccination and to monitor its impact on the epidemiology of HBV infection in a highly endemic area in sub Saharan Africa.

Despite different vaccination regimes, vaccine efficacy (VE) against chronic infection remains high in this population (94–96%) [16]–[19] although infection defined by the sole presence of hepatitis B core antibody (anti-HBc) have occurred in vaccinated subjects [16]. These infections increased with age and time since vaccination ranging from 2–3% in young children to 20–30% in persons >20 years old [17], [18]. In some cases the infections were transient but in others in whom anti-HBc persists it is not known if the virus is present in occult form.

Here we report the result of the 6th survey that was conducted between 2008 and 2009. We determined vaccine efficacy against infection and chronic infection and concentrated on antibody decay, risk factors for HBV infections including marriage and molecular monitoring of possible occult infections.

## Methods

### Ethical approval

The study was approved by the joint Gambia Government/MRC Unit and the London School of Hygiene and Tropical Medicine Ethics Committees.

### Subjects

The Keneba-Manduar study is an open community cohort study of HBV vaccine efficacy, which has conducted five serial cross-sectional surveys at approximately 4–5 year intervals (1984, 1989, 1993, 1998, 2003); the methods for these surveys have been described previously [17]. In 1984, all non-immune children <5 years were vaccinated against HBV in a trial of 3 regimens of plasma derived hepatitis B vaccine. Since that time routine vaccination of infants has been undertaken among all children born in the villages. During each survey, an assessment of HBV seromarkers was carried out in cohort members. These seromarkers included HBV core antibody (anti-HBc), HBV surface antibody (anti-HBs) and HBV Surface Antigen (HbsAg). Information on peak response to vaccination was measured two months after infant vaccination and was collected in the study database.

In the current study conducted from 2008–2009, adults and children aged >1 year who had received a HBV vaccination during infancy (or at <5 years if non-immune and vaccinated in 1984), who resided in the village of Keneba or Manduar were eligible for inclusion. Eligible participants were identified from a database containing records of all participants of previous Keneba-Manduar cross-sectional survey as well as the database of infants vaccinated between the 2003 and the present survey. The database includes information on West Kiang DSS number, name, date of birth, HBV vaccination dates, HBV seromarkers, blood collection dates from previous Keneba-Manduar surveys, marital status and identifying information on parents and, where relevant, spouse/s. After signed informed consent was obtained from the mother of a child or from an adult blood was collected. In the case of children <2 years of age this was by fingerprick (1.0 mL) and in the case of children 2 yrs (or older) and adults, by venepuncture (2.0–5.0 mL).

The type of HBV vaccine, the manufacturer of vaccine and schedule of administration has varied over time. This has included intradermal vaccine, 3 or 4 doses of plasma-derived vaccine and 3 doses of recombinant vaccines [16]. Since the previous survey in 2003 until 2009 three doses of recombinant vaccine have been given. Vaccines were provided by the Expanded Programme of Immunisation (EPI) and included those made by Shantha Biotech, India (1993–2005); Heber Biotec-Cuba (2005–2007) and by Green C vaccine Corp-Korea (2007). A pentavalent vaccine containing HBV recombinant vaccine (Easy Five ^TM^ – Pinacea Biotec, India) was introduced in April 2009, and subsequently the vaccination regime changed to one single dose of recombinant HBV vaccine at or soon after birth followed by 3 doses of pentavalent vaccine at 6,10 and 14 weeks. To ensure vaccination, infants and children are invited to ‘call clinics’ operated by MRC, Keneba and those who do not turn up after the first call are re-called to attend the following week until the vaccination is issued.

### Hepatitis B virus serology

All laboratory work was performed at the MRC Unit The Gambia. Antiboby against hepatitis B surface antigen (anti-HBs) and antibody against hepatitis B core antigen (anti-HBc) was measured by Elisa (DiaSorin). Samples positive for anti-HBc were tested for hepatitis B surface antigen (HBsAg by immunochromatography (Abbot Determine™) and HBV DNA by quantitative PCR (qPCR). HBsAg positive samples were further tested for Hepatitis B e antigen (HBeAg) and antibodies (anti-HBe) by Elisa (DiaSorin, Sallugia, Italy) and for HBV DNA by quantitative PCR (qPCR).

Seromarkers used in analysis were defined as follows: chronic infection was defined as the detection of HBsAg on two occasions at least 6 months apart. Breakthrough infection was defined as anti-HBc seropositivity in vaccinated subjects who were not chronically infected. HBV infection was defined as either of the above. Undetectable anti-HBs was defined as <10 (mlU/mL). Primary non-response to vaccination was defined as a peak response of <11 (mlU/mL). Subjects with missing peak response data who had detectable anti-HBs in a subsequent survey were categorized as primary responders.

### HBV DNA quantification

DNA was extracted from HBsAg positive and anti-HBc positive samples using QIAamp DNA Mini Kit (Qiagen, UK) and quantified using real time PCR with HBV specific primers as previously described and utilizing primers HBV TAQ 1 (GTG TCT GCG GCG TTT TATCA) and HBV TAQ-2 (GAC AAA CGG GCA ACA TAC CTT) for the amplification [20]. The sensitivity of the assay was 200 copies/ml.

### Data collection

Following community approval, trained field staff identified eligible subjects by verifying name, date of birth and village as well as either the spouse, mother or father names using study data cards pre-populated with participant information. Subjects were informed about the study and invited to participate. If they accepted, a consent form was administered; subject consent (or parental consent on behalf of a minor) was taken as either a signature or oral consent which was witnessed by the field worker. Blood specimens were collected and stored in cool box during field work, and were processed within 5 hours of collection. Data collection was carried out in two phases. Initially, data collection was staged in the villages of Keneba and Manduar over a 2 week period during November 2008, during which time 58% of the study participants were enrolled. There is a high rate of internal migration from inland villages to the coastal areas of The Gambia. Follow-up of participants who were not found during the initial phase was carried out over a nine month period from January – July 2009.

### Statistical analysis

An SQL database of participant information and HBV seromarkers from previous Keneba-Manduar surveys was maintained at MRC in Fajara. Participant information from the current survey was entered, and HBV seromarkers data from the laboratory were uploaded into this database. The final data set thus included HBV seromarker data on participants from up to six time points (1984, 1989, 1993, 1998, 2003 and 2008).

In the Kaplan-Meier analysis assessing probability of detectable anti-HBs, date of anti-HBs was defined as the blood draw date from 2008 survey. Subjects with detectable anti-HBs were censored. Survival time was calculated as the time since the subject's last HBV vaccination in the series and the date of undetectable anti-HBs or the censor date, in subjects with and without undetectable anti-HBs, respectively. Subjects who were missing data on category of primary response, were primary non-responders or who had missing anti-HBs data for 2008, were excluded from the analysis. One subject who was a primary responder but identified as chronically infected in 2008 was also excluded. In the analysis of Geometric Mean anti-HBs titre (GMT) by time since vaccination, GMTs were calculated within categories defined by the combination of age group and peak response. Age groups were defined as 1–4 year, 5–9 years, 10–14 years, 15–19 years, 20–24 years and 25–29 years. The GMT analysis of decay of anti-HBs was restricted to study subjects with data for all surveys in which they were eligible. Anti-HBs values at follow-up which were <11 were set at 1. In the Kaplan-Meier analysis of time to HBV infection, date of infection was defined as the blood draw date from the first survey in which the subject was anti-HBc positive. Subjects with no record of HBV infection in any survey were censored on the blood draw date of their most recent survey. Survival time was calculated as the time since the subject's last hepatitis B vaccination in the series and the infection date or the censor date, in infected and censored subjects respectively. In subjects with HBV infection, infection was estimated as occurring at the mid-way point between surveys, or between HBV vaccination and subsequent survey in subjects who participated in only one survey. A Kaplan-Meier analysis of time to HBV infection by marital status was carried out in women age 14 or above, where marital status was defined dichotomously as ‘ever married’ versus ‘never married’. Women entered the analysis from the date of their 14th birthday; those already infected with HBV by age 14 were excluded from analysis. Marital status was treated as a time-varying factor participants could contribute to more than one marital status strata and entered the ‘married’ strata on their earliest date of marriage; married woman who subsequently divorced or were widowed were retained in the ‘married’ strata.

In the analyses of vaccine efficacy (VE) against HBV infection and chronic infection, vaccine efficacy was calculated as 1– (prevalence among vaccinated subjects in 2008/prevalence among unvaccinated subjects). Five children who became infected during the initial primary vaccination program in 1984 were excluded from analyses of vaccine efficacy.

The age-specific prevalence of HBV infection and chronic infection in Keneba and Manduar from 1984 prior to the introduction of HBV vaccination was used as the prevalence among unvaccinated subjects [5]. In 1984, the prevalence of HBV infection and chronic infection remained stable in the population from age 15 onwards; in the current analysis prevalence in 15–19 year olds from the 1984 survey was used as a baseline for all adults. Thus in our analysis of VE it was assumed that the age-specific prevalence of seromarkers in 2008 reflected cumulative incidence of anti-HBc seroconversion and chronic infection within each age category. We ran a logistic regression model of chronic infection on a binary vaccination status (vaccinated versus unvaccinated) for each combination of age group and peak response category to obtain estimates of VE and 95% confidence intervals (CI) for VE. The upper and lower limits of CIs were calculated from the exponentiated values of the linear sum of model parameters. Logistic regression was also used to obtain odds ratios (ORs) for the risk of infection associated with age group, sex, peak response category and village.

## Results

A total of 1900 subjects aged 1–29 years and resident (or originally resident) in Keneba and Manduar were identified as eligible for follow-up in this survey. Of these, a total of 392 (20.6%) were excluded from the analyses either because there was no record of immunization during infancy (or <5 years if immunized in 1984) with 3 or more doses of vaccine (n = 342) or the doses were given at <28 days apart (n = 50). Among the remaining 1508 subjects targeted for the final sample, 1276 (84.6%) were successfully followed up, 118 (7.8%) refused participation, 94 (6.2%) were not located, and 20 (1.3%) had died. Study retention was significantly higher among residents of Manduar (91.8%) versus Keneba (82.2%) and higher in women (88.8%) than men (80.8%). There was also a significant trend for higher retention in younger versus older age groups; the highest retention was found in 5–9 year olds (91.7%) and the lowest in 25–29 year olds (74.7%).

### Antibody to Hepatitis B Surface Antigen

**Figure 1:**
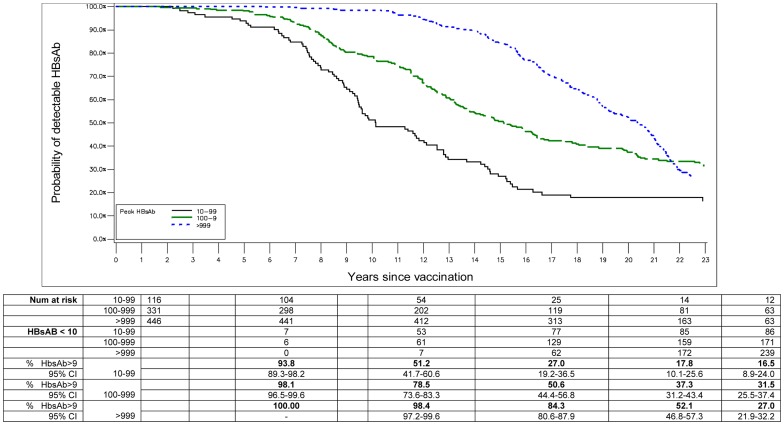
Probability of remaining anti-HBs positive by time since vaccination and peak response for HBsAb. Table shows number at risk, number of subjects with undetectable HBsAb (HBsAb <10), and the percent of subjects with detectable HBsAb (HBsAb>9) at 0, 5, 10, 15, 20 and 23 years post-infant vaccination, by HBsAb peak response category (10–99, 100–999, >999).

**Figure 2:**
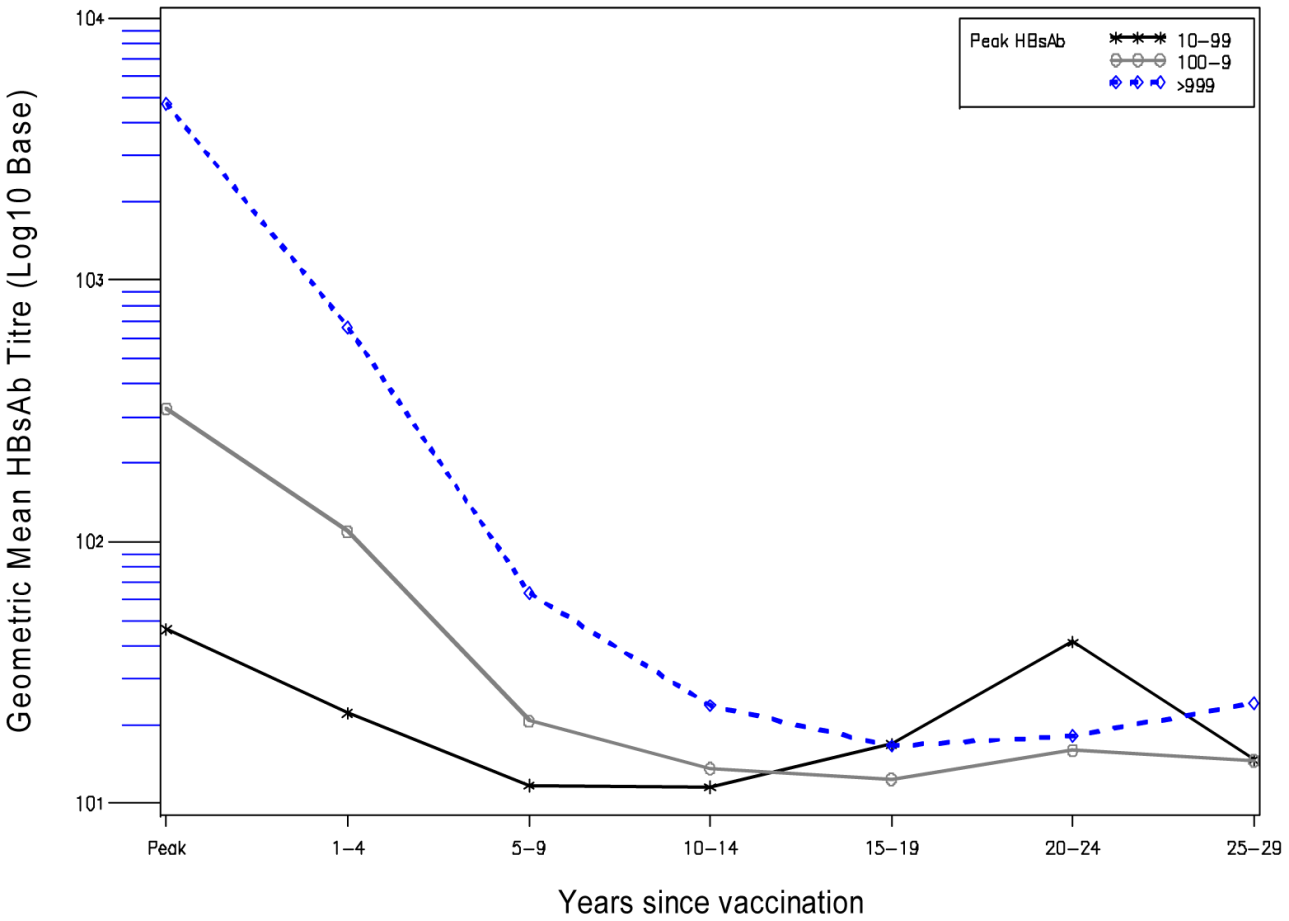
Decrease in antibody to hepatitis B surface antigen (anti-HBs) by time since vaccination, and peak anti-HBs response (mlU/mL).

Of 1508 participants, 176 were missing peak response data (and did not have detectable anti-HBs in a subsequent survey with which to identify them as primary responders). Of the 1332 participants with data which to assess peak response, 1218 (91.4%) were primary responders and 114 (8.6) were primary non-responders. In Kaplan-Meier analysis, the probability of remaining HBs antibody positive varied significantly by peak response category (log rank P<0.0001, Figure 1). In vaccinated subjects in the lowest peak bleed response category of ‘10–99 (mlU/mL)’, the probability of detectable HBs antibody at 5, 10, 15, 20 and 23 years, was 93.8%, 51.2%, 27.0%, 17.8% and 16.5% respectively. Vaccinated subjects in the highest peak bleed response category of ‘>999 (mlU/mL)’, had a significantly higher probability of detectable anti-HBs at all time points. At 5, 10, 15, 20 and 23 years these probabilities were 100.0%, 98.4%, 84.3, 52.1%, and 27.0% respectively. Figure 2 shows that in all peak response categories GMT for anti-HBs falls steeply up to nine years past vaccination and plateaus to a low level past year nine. It is interesting to note that there is a slight upward trend in GMT for anti-HBs at and beyond fifteen years post-vaccination, which is most pronounced in the lowest peak response category used in the analysis (10–99 (mlU/mL). However this should be interpreted with caution because of small numbers. In a logistic regression model that included age, log peak response, sex, and village, previous break through infection was associated with a doubling of odds of having detectable hepatitis surface antibody in the current survey (OR = 2.1, 95% CI: 1.4, 3.1, (analysis not shown).

### HBV Infection

There were 1251 subjects with data on anti-HBc, among whom 127 (10.2%) had HBV infection (Table 1). A further 40 (3.6%) had transient ant-HBc seroconversion in a previous survey but were currently anti-HBc negative; 2 more subjects were persistently anti-HBc positive over 2 consecutive surveys but had cleared the infection and were anti-HBc negative in 2008. In logistic regression analysis, several factors were independently associated with increased odds of current HBV infection. These factors were older age, male gender and lower peak response category. Current HBV infection was also marginally associated with being a resident of Manduar village compared to Keneba (Table 2).

**Table 1:** Frequency of HBV infection and chronic infection in 2008, by age group and village, among all vaccinated subjects (regardless of peak bleed).

Variable	No (%) of HBV infections	Adjusted Odds Ratio (95% CI)	P – value for variable
*HBV infection*			
Age	Keneba	Manduar	Keneba and Manduar
1–4 yr	5/106 (4.7)	1/43 (2.3)	6/149 (4.0)
5–9 yr	4/205 (1.9)	3/70 (4.3)	7/275 (2.6)
10–14 yr	12/208 (5.8)	7/81 (8.6)	19/289 (6.6)
15–19 yr	10/164 (6.1)	8/65 (12.3)	18/229 (7.9)
20–24 yr	23/126 (18.3)	18/70 (25.7)	41/196 (20.9)
25–29 yr	29/93 (31.2)	7/20 (35.0)	36/113 (31.9)
Total	83/902 (9.2)	44/349 (12.6)	127/1251 (10.2)
Age		Chronic infection	
1–4 yr	0/105 (0.0)	1/43 (2.3)	1/148 (0.7)
5–9 yr	2/205 (1.0)	0/70 (0.0)	2/275 (0.7)
10–14 yr	3/208 (1.4)	1/80 (1.3)	4/288 (1.4)
15–19 yr	0/163 (0.0)	1/65 (1.5)	1/228 (0.4)
20–24 yr	1/124 (0.8)	1/69 (1.5)	2/193 (1.0)
25–29 yr	2/91 (2.2)	1/20 (5.0)	3/111 (2.7)
Total	8/896 (0.9)	5/347 (1.4)	13/1243 (1.1)

**Table 2:** Risk factors for HBV infections.

Variable	No (%) of HBV infections	Adjusted Odds Ratio (95% CI)	P –value for variable
*Age in years:*			
1–4	4/87 (4.6)	1.0	
5–9	6/192 (3.1)	0.6 (0.2, 2.3)	
10–14	16/234 (6.8)	1.7 (0.5, 5.4)	
15–19	18/218 (8.3)	2.6 (0.8, 8.4)	
20–24	41/196 (20.9)	7.9 (2 6, 24.1)	
25–29	36/113 (31.9)	14.2 (4.7, 43.4)	<.0001
*Sex*			
Female	56/530 (10.6)	1.0	
Male	65/510 (12.8)	1.5 (1.0, 2.3)	0.0378
Peak anti-HBs (mlU/mL):			
>999	47/461 (10.2)	1.0	
100–999	33/362 (9.1)	1.3 (0.8, 2.2)	
10–99	18/122 (14.8)	3.9 (2.0, 7.6)	
<10	23/95 (24.2)	3.7 (2.0, 6.9)	<.0001
Village:			
Keneba	78/742 (10.5)	1.0	
Manduar	43/298 (14.4)	1.6 (1.0, 2.4)	0.0442

The analysis includes 1040 subjects; 211 out of the 1251 subjects were missing information on peak bleed.

**Figure 3:**
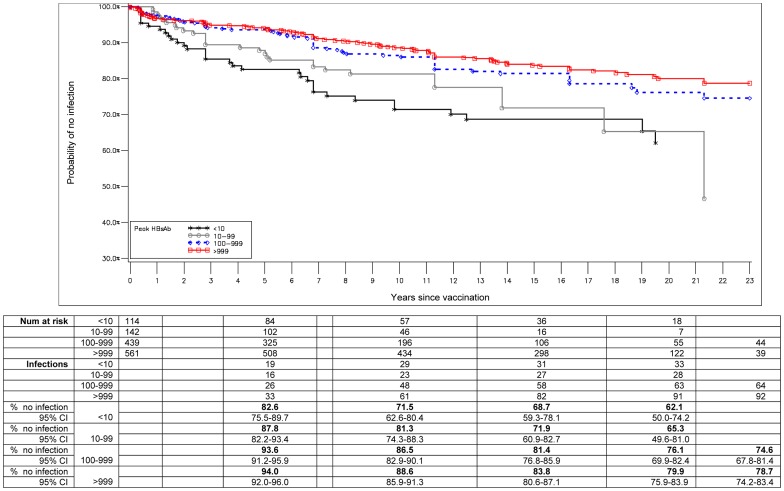
Probability of remaining uninfected (as determined by lack of anti-HBc) by time since vaccination and peak response for anti-HBs (1256 individuals). Table shows number of subjects at risk, number of subjects with HBV infection, and the percent of subjects remaining uninfected at 0, 5, 10, 15, 20 and 23 years post-infant vaccination, by HBsAb peak response category (<10, 10–99, 100–999, >999).

A total of 1256 participants with data on peak response and anti-HBc status at survey timepoints were included in a Kaplan-Meier analysis of HBV infection, among whom 219 (17.4%) HBV infections occurred including 13 chronic infections. This analysis included subjects from both the 2008 survey as well as subjects who participated in past surveys. The probability of remaining uninfected varied significantly by peak response category (log rank test P<0.0001) (Figure 3). Among participants in the highest peak response category of >999 (mlU/mL), the probability of remaining uninfected was 94.0%, 88.6%, 83.8% and 79.9% at 5, 10, 15, and 20 years, respectively. This compares to the probability of remaining uninfected of 87.7%, 81.3%, 71.9%, and 65.3% at 5, 10, 15, 20, respectively in the peak response category of 10–99 (mlU/mL). Infection-free survival did not vary significantly between lowest peak response category of ‘10–99 (mlU/mL)’ and non-responders (analysis not shown).

### Hepatitis B virus DNA and HBV Infection

113 out of the 127 individuals with HBV infection were tested for HBsAg (14 missing due to small volume of blood sample) and all 113 were tested for HBV DNA; 13 were positive for HBsAg and 6 of those with chronic infections tested positive for HBV DNA (one result missing). None of the remaining 100 anti-HBc positive individuals who were negative for HBsAg tested positive for HBV DNA.

### Hepatitis B virus infection and marriage

There were 331 women aged 14 years or above in the 2008 survey, of whom 302 had complete marital status data available. In total, 52 children who were infected with HBV before the age of 14 years were excluded from the analysis. Of the remaining 250 women included in the analysis, 127 were not married in whom 1 HBV infection occurred. In the 123 women who were married, 11 HBV infections occurred, of which 3 occurred before marriage and 8 occurred after marriage. Eight out of the 11 were tested for HBsAg and they were all negative. The log-rank test for equality of survivor functions, showed no significant difference in time to HBV infection by marital status (Log-rank p-value = 0.1014).

### Chronic hepatitis B virus infection

Of 1276 participants followed in the current survey 1243 (97.4%) participants had data with which to assess chronic infection status, of whom 13 (1.0%) had positive HBV surface antigen (HBsAg) (Table 1). Of these, 10 had been identified as chronic carriers in a previous survey and 3 were newly identified. The newly identified cases were not yet re-tested for HBsAg. Among these 13 carriers (aged 3 to 28 years old), 9 were primary non-responders, 3 were primary responders and 1 was missing primary response data. The three primary responders were identified as chronic infections between the ages of 7–10 years. All the chronic infections had received at least 3 doses of HBV vaccine and HBeAg positivity persisted for up to 15 years in the older carriers.

Nine mothers of the 13 chronic infections were tested in the surveys preceding the delivery of their children, all except one tested positive for HBsAg and HBeAg. Thus it is likely that many of the 13 children were infected at the time of delivery or soon after birth before HBV vaccine was given.

In addition to these 13 chronic infections, there were a further 9 participants who had been identified as chronic infection in a previous survey but who had converted to HBsAg negative status at the time of the current survey. In logistic regression analysis of chronic infection on age, sex, primary responder status and village, being a primary non-responder was significantly associated with risk of HBV carriage (OR: 31.5, 95% CI: 8.3–117.8) (analysis not shown).

### Vaccine Efficacy

In the study population, overall vaccine efficacy against chronic infection and HBV infection was 95.1 (95% CI: 91.5–97.1) and 85.4 (82.7–87.7), respectively (Table 3). Vaccine efficacy against chronic infection did not vary significantly between villages or by age group. Vaccine efficacy against HBV infection also did not vary significantly between villages, but tended to fall with age (P for linear trend = .046) and was significantly lower in those 20 years or older compared to younger age groups (P<.0001).

**Table 3:** Vaccine efficacy against HBV infection and chronic infection in 2008 by age group and village, among all vaccinated subjects (regardless of peak bleed).

HBV infection			
*Age*	*Keneba*	*Manduar*	*Keneba and Manduar*
1–4 yr	82.9 (59.7–92.7)	96.3 (74.4–99.5)	89.8 (77.6–95.3)
5–9 yr	96.4 (90.6–98.6)	94.9 (84.7–98.3)	96.1 (91.8–98.1)
10–14 yr	94.3 (89.2–97.0)	92.1 (82.9–96.3)	93.6 (89.5–96.1)
15–19 yr	92.6 (86.5–96.0)	92.6 (86.5–96.0)	91.7 (86.8–94.7)
20–24 yr	78.7 (68.9–85.4)	78.7 (68.9–85.4)	77.5 (70.3–83.0)
25–29 yr	64.1 (50.7–73.8)	51.4 (27.5–67.5)	66.5 (55.4–74.8)
Overall	84.4 (80.7–87.4)	86.0 (81.3–89.6)	85.4 (82.7–87.7)
**Chronic infection**			
*1–4 yr*	*100.0 (Inestim.)*	*93.7(56.4–99.1)*	*96.5 (75.3–99.5)*
5–9 yr	93.7 (75.2–98.4)	100.0(Inestim.)	96.6 (86.5–99.1)
10–14 yr	89.8 (68.5–96.7)	96.5 (75.8–99.5)	93.2 (82.1–97.4)
15–19 yr	100.0 (Inestim.)	95.4 (67.7–99.3)	98.1 (86.8–99.7)
20–24 yr	95.4 (67.4–99.3)	95.8 (70.4–99.4)	95.7 (82.8–98.9)
25–29 yr	87.8 (51.8–96.9)	88.0 (18.1–98.2)	89.4 (67.6–96.5)
Overall	93.6 (87.3–96.8)	95.9 (90.3–98.3)	95.1 (91.5–97.1)

## Discussion

The main finding from this study is that hepatitis B vaccine given in infancy or early childhood in rural Gambia provides excellent protection against HBV chronic infection for at least 24 years. The major risk factors for the few persons who became carriers were an undetectable primary antibody response and a mother who was HBeAg positive. In contrast protection against anti-HBc seroconversion declined markedly with time being dependent on initial peak antibody response, age and sex. Marriage did not significantly increase the risk of infection for women. HBV DNA was not detected in any HBsAg negative individual with HBV infection. Hepatitis B antibody concentrations decayed rapidly over the first nine years after vaccination then flattened to a low level: 20 years after vaccination the majority of vaccinated villagers had no detectable antibody. Persons who converted to ant-HBc antibody positive were more likely to have detectable concentrations of antibody suggesting a booster effect.

This is one of longest community based studies of efficacy of hepatitis B vaccine ever reported. The cohort size is large and follow up has been remarkably good though loss of follow up increases as adolescents and young adults migrate to urban areas seeking further education or work. It is one of the very few studies which had measured peak antibody response for each vaccinated person. This measurement has proved to be a very informative immunological correlate of protection against both anti-HBc seroconversion and chronic infection.

Interestingly there is a marginal difference in the level of anti-HBc positivity in vaccinated individuals from the two study villages;. The rates are higher in Manduar and this is similar to the findings prior to the introduction of the vaccine. There is no clear reason for the observed differences between the two neighbouring villages with similar environmental factors, practices and ethnicity.

Although numbers are small and we have insufficient data on the hepatitis B serostatus of the male spouse (they were unavailable to have blood test) it is the first to study the risk of marriage for HBV infections in vaccinated individuals. This is important as in The Gambia and in other West African countries the male spouse is likely to be unvaccinated, 5–10 years older [21] and with a 10–15% likelihood of being a chronic carrier [5].

Although historical control data were used to estimate vaccine efficacy, the prevalence of infected persons and chronic infections is similar as observed in subsequent studies of unvaccinated children at different ages in other regions of The Gambia [22], [23]. In a different study, of hepatitis B vaccine efficacy conducted in the coastal region in 2004 unvaccinated 15 year old adolescents had a prevalence of HBV infection of 53% and chronic infection of 13% [24].

Different vaccines and different regimes were used at different periods of the study; thus direct comparison of vaccine efficacy for each regime was not possible. The sensitivity of our HBV DNA test was not of the highest order so we are unable to claim complete absence of virus following anti-HBc seroconversion which can only be verified by liver biopsy and more sensitive PCR assays.

Other long term longitudinal studies of hepatitis B vaccine efficacy have also been conducted in Alaska [25] Taiwan [26], [27] Thailand [28] China [29] and in The Gambia [30]. The latter differs from the current study in that it is a randomised nationwide study but lacks data on peak antibody responses in the later years of follow-up [31]. Similarities between the current study and those previously published have been noted although the level of endemicity and epidemiology of HBV infection has varied with region: vaccine induced antibody decays with time, anti-HBc seroconversion occurs in those with low antibody concentrations, efficacy against chronic infection is maintained and the incidence of acute hepatitis and hepatocellular carcinoma drops dramatically [14], [32]. Only the current study from the Gambia and the Alaskan study which vaccinated all persons greater than 6 months of age has related peak antibody responses to long term outcome which has proved such an important predictor in the current analysis. The Asian studies differ as children of HBeAg positive mothers, which is common in the region received Hepatitis B immunoglobulin together with the first dose of hepatitis B vaccine at birth. In Taiwan although break through infections decreased with time a high proportion were due to mutant viruses which resulted in occult infection as defined by anti-HBc and HBV in serum [33]. In The Gambia 15 years after the start of nationwide infant hepatitis B vaccination and despite widespread circulation of the virus vaccine escape mutants were not detected in vaccinated HBV chronic infections [34]. Anti-HBc seroconversion may persist or clear in time resulting in a small boost in antibody concentrations as has also been observed in exposed but uninfected vaccinees in the Alaskan study [35] and the current study. Whether these transient infections are necessary to boost cell mediated immunity is unknown. We noted that males are more susceptible to anti-HBc seroconversion and in the past before the advent of vaccination they had a higher risk of chronic hepatitis B carriage [11] and of hepatocellular carcinoma [9]. Marriage is a risk factor for sexually transmitted infections in The Gambia [36]. In this predominantly Muslim society the husband is often much older than the wife [21], is more likely to be a carrier and less likely to have been vaccinated against hepatitis B virus as the national program only started 20 years ago. This potential risk factor for transmission in vaccinated populations deserves further investigation in a large study.

The prevalence of HBeAg positive mothers is relatively low in The Gambia compared to Asia yet a very high proportion of chronic carriers in this study were children of such mothers. Most failed to respond to vaccination either because the response was masked by infection acquired from mother before or during hepatitis B vaccination or possibly as the result of a maternally linked genetic defect [37], [38].

The major finding of this paper is that in a rural community with a high level of endemic hepatitis B infection vaccination of infants has been able to prevent chronic infection for at least 24 years. Protection against carriage was not directly related to peak antibody response apart from a few persons who failed to respond to the vaccine. However anti-HBc seroconversions which correlated with peak antibody response and time since vaccination increased markedly in late adolescence. This may have resulted from sexual exposure or due to migration to urban areas where vaccination has been more recent and exposure to hepatitis B virus is greater. Important questions are whether these infections persist in occult form causing liver damage or whether they are beneficial, thus boosting humoral and cellular immunity. The first possibility is thought unlikely as plasma HBV DNA was not detected in any of the persons with breakthrough infection but can only be ruled out after histological and virological examination of liver biopsy. The second possibility is supported by a modest increase in serum antibody but further research is needed to show how long antibody persists and that cellular immunity is boosted. A booster dose of vaccine given to seropositive or seronegative persons in Thailand 5 years after vaccination resulted in similar rises in antibody concentrations which decayed with time but persisted for 15 or more years [32]. In The Gambia the rise in HBs antibody following a boost at 15 years of age was lower and fell markedly over the next year [24]. In Taiwan children of HBeAg positive mothers showed a marked antibody response to a booster dose of vaccine at 5 years of age but little change in cellular immunity [33]. However we could find no convincing evidence from highly endemic areas that boosted persons have less anti-HBc seroconversions or are less prone to chronic infection. More proof of improved efficacy is needed before a booster dose of vaccine is recommended in such populations [27].

Many studies of hepatitis B vaccine have shown that a small proportion of the population are genetically incapable of mounting an adequate immune response to the vaccine and are at high risk of becoming carriers [37], [39], [40]. In our study most children who became persistently infected had an undetectable response to the vaccine and their mothers also tested HBeAg positive which suggests a genetic link. However we were unable to distinguish if antibody responses might have been masked by early infection. The majority of children of HBeAg positive mothers can be partially protected by Hepatitis B vaccine given immediately after birth [41]. WHO currently recommends the first dose of vaccine is given as early as possible after birth but unfortunately in rural areas this is often delayed and in many countries the first dose is given in combination with other EPI vaccines at 1 or 2 months of age. It has been suggested that BCG enhances antibody and cellular responses to hepatitis B vaccine and when given at birth diminishes neonatal mortality [42], [43]. Both vaccines should be given as early as possible after birth.

## Conclusions

Gambia infants given hepatitis B vaccine in infancy are well protected in childhood but are at increased risk of hepatitis B infection, but not chronic infection, in adolescence and early adulthood. Married women were not shown to be at increased risk. There is no compelling evidence of the need for a booster dose of vaccine in The Gambia but further research is required to determine if breakthrough viral infections signified by anti-HBc seroconversion only persist in the liver and cause harm or whether they are beneficial by boosting immunity. Hepatitis B vaccine should be given with BCG vaccine immediately after birth to prevent early hepatitis B infection.
